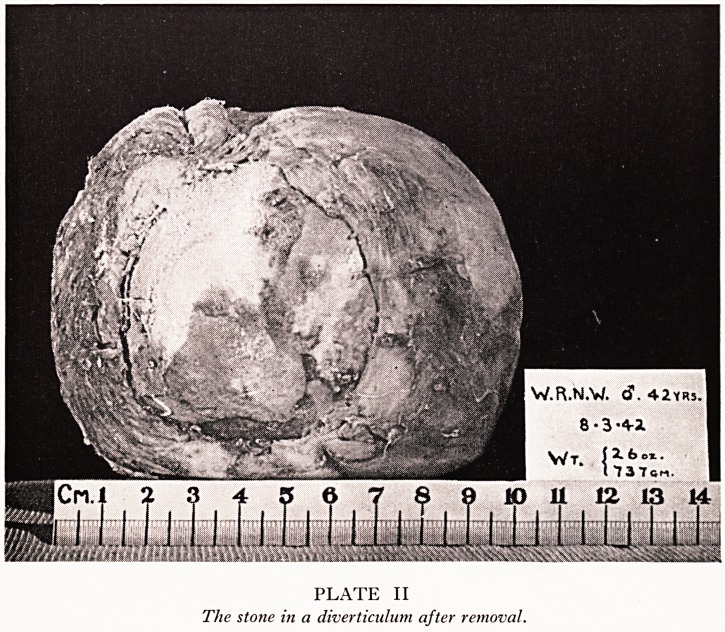# Twenty Year Follow-Up of a Case of Massive Calculus in a Vesical Diverticulum

**Published:** 1964-01

**Authors:** A. Wilfrid Adams


PLATE I

*Radiograph of the stone* in situ.

PLATE II

*The stone in a diverticulum after removal.*

### TWENTY YEAR FOLLOW-UP OF A CASE OF MASSIVE CALCULUS IN A VESICAL DIVERTICULUM

BY

A. WILFRID ADAMS, M.S., F.R.C.S.

(At a meeting of the Bristol Medico-chirurgical Society in October 1962 there was exhibited a specimen, now in the Southmead Hospital Museum, of a massive calculus lying in a diverticulum of the bladder. This had been removed at operation in 1942 by Mr. Adams, who reported the case at that time (1943). Mr. Adams has now contributed the following further account of the patient.—Ed.)

Because of the encouraging sequel to this singular venture a further record seems called for. The calculus weighed 737 grams. Searching the literature on this specific category I found its nearest rival weighed 300 grams (Rathbun, 1924). Twenty years have now elapsed since the patient was relieved of this burden, and so far his urinary health remains normal.

When seen at the age of 42 years the patient had dysuria, and had passed a stone in childhood. Later, symptoms of lower abdominal discomfort had developed and sometimes severe pains. In 1929 he was disabled for some time by cystitis and haematuria. In 1930 he was seen by Mr. F. Kidd who extracted a stone from the terminal urethra; after cytoscopy Mr. Kidd advised excision of a vesical pouch but the patient declined. During the ensuing decade he passed more stones and had nocturia—three or four times a night—but no frequency by day. He was constipated.

In 1942 his illness culminated in an attack of cystitis, right renal colic, and haematuria. Until 3 weeks before this he had been actively engaged in the Home Guard. On examination his general appearance was fair, there was tenderness in the right loin, and the pelvis was filled by a smooth, hard, rounded, and fixed swelling. This, on skiagraphy, proved to be a calculus (Plate I). Pyelography showed a large right hydronephrosis.

At operation the calculus (Plate II) in its diverticulum was removed by transverse incision of the rectus sheath, traction with obstetric forceps suprapubically, and digital and instrumental pressure *per rectum*. It was the size of a foetal head at full term. Recovery was smooth.

In 1943, his symptoms gone, the patient was on full duty with the Home Guard. His bowels acted well without aperients. A right pyelogam was normal and cystoscopy revealed a ridge of scar tissue at the site of the former pouch. Residual urine was 8 ounces. In 1960 he was seen by Dr. J. M. Naish for hypertension; there was no uropathy. In 1963 he chatted cheerfully with me and told me his urinary functions were normal.

(The illustrations are reproduced by kind permission of the British Journal of Urology.)

### REFERENCES

Adams, A. W. (1943). *Brit. J. Urol.*, **15**, 136.

Rathbun, N. P. R. (1924). *J. Urol.*, **1**, 12.

## Figures and Tables

**PLATE I f1:**
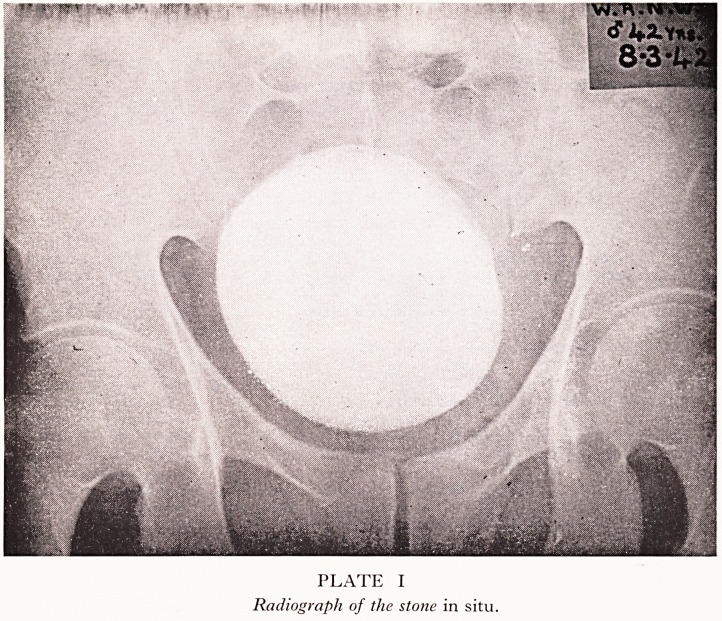


**PLATE II f2:**